# Ravens Reconcile after Aggressive Conflicts with Valuable Partners

**DOI:** 10.1371/journal.pone.0018118

**Published:** 2011-03-25

**Authors:** Orlaith N. Fraser, Thomas Bugnyar

**Affiliations:** 1 Department of Cognitive Biology, University of Vienna, Vienna, Austria; 2 Konrad Lorenz Forschungstelle, Grünau, Austria; University of Lethbridge, Canada

## Abstract

Reconciliation, a post-conflict affiliative interaction between former opponents, is an important mechanism for reducing the costs of aggressive conflict in primates and some other mammals as it may repair the opponents' relationship and reduce post-conflict distress. Opponents who share a valuable relationship are expected to be more likely to reconcile as for such partners the benefits of relationship repair should outweigh the risk of renewed aggression. In birds, however, post-conflict behavior has thus far been marked by an apparent absence of reconciliation, suggested to result either from differing avian and mammalian strategies or because birds may not share valuable relationships with partners with whom they engage in aggressive conflict. Here, we demonstrate the occurrence of reconciliation in a group of captive subadult ravens (*Corvus corax*) and show that it is more likely to occur after conflicts between partners who share a valuable relationship. Furthermore, former opponents were less likely to engage in renewed aggression following reconciliation, suggesting that reconciliation repairs damage caused to their relationship by the preceding conflict. Our findings suggest not only that primate-like valuable relationships exist outside the pair bond in birds, but that such partners may employ the same mechanisms in birds as in primates to ensure that the benefits afforded by their relationships are maintained even when conflicts of interest escalate into aggression. These results provide further support for a convergent evolution of social strategies in avian and mammalian species.

## Introduction

Aggressive conflict features regularly in the lives of group-living animals but may entail significant costs, including loss of time and energy, risk of injury, damage to the opponents' relationship and post-conflict distress [1]. Reconciliation, a post-conflict affiliative interaction between former opponents [2], may mitigate such costs through distress alleviation and relationship repair [3]. Reconciliation is not expected to occur after all conflicts, but only when former opponents share a valuable relationship, as for such partners the value of reinstating benefits afforded by the relationship should outweigh the risks of renewed aggression upon approaching a former opponent ['valuable relationships hypothesis'; 4,5–8].

Reconciliation has been demonstrated in many primates [3] and a few other mammalian species [9]–[13], but, despite two attempts [14], [15], reconciliation has never been demonstrated in birds. The absence of reconciliation in birds may result from a general difference in avian and mammalian behavior as the fluidity of avian social systems may facilitate post-conflict dispersal. Alternatively, the pair-bonded nature of most bird species may preclude the need for reconciliation as pair partners rarely engage in aggressive conflict [16] and other partners may not share a relationship of sufficient value to merit reconciliation. However, if valuable relationships do exist outside the pair bond, as has been recently shown for a group of subadult ravens [17], those birds may employ similar conflict resolution mechanisms to primates and other mammals and reconciliation may occur.

In this study, we investigated the occurrence of reconciliation in another group of captive subadult ravens. We further examined the influence of conflict intensity and opponent relationship quality on reconciliation, predicting that reconciliation may be more likely to occur after more intense conflicts as a result of increased post-conflict distress and that conflicts between valuable partners would be most likely to be reconciled as the benefits of relationship repair would be higher for such partners. Finally, we investigated the interdependency between reconciliation and renewed aggression between former opponents, predicting that if post-conflict affiliation between former opponents in birds serves the same relationship repair function as has been demonstrated in primates, the likelihood of renewed aggression should be lower following reconciliation than if reconciliation does not occur.

## Results

### Do ravens reconcile?

**Figure 1:**
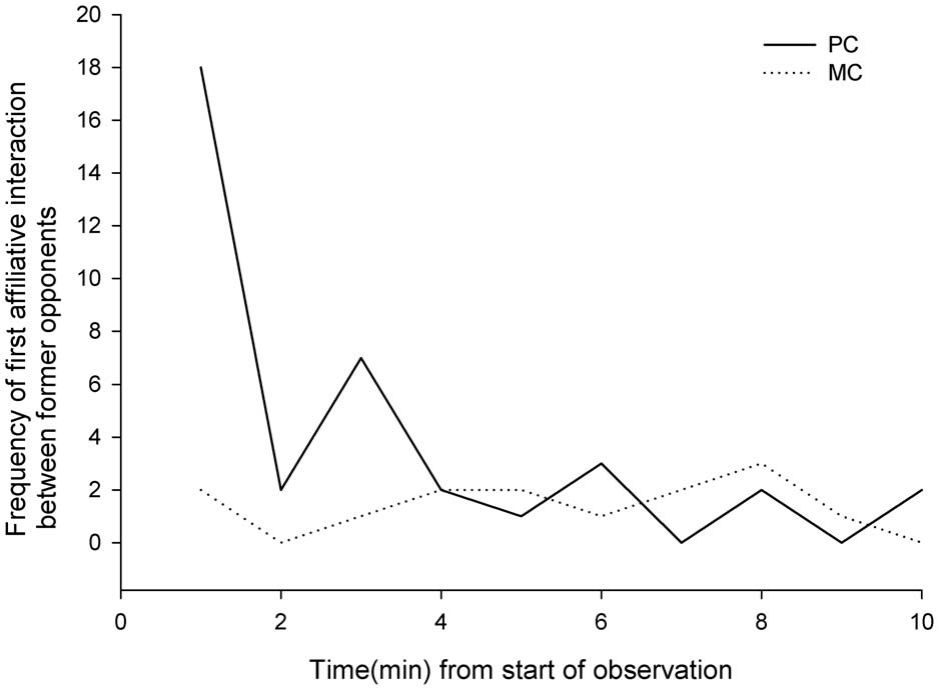
Latency to first affiliative contact between former opponents in the ten minutes following aggression (post-conflict observations; PC) and during matched control observations (MC).

We demonstrated the occurrence of post-conflict reconciliation in a group of seven captive ravens by showing that the latency to first affiliative contact between former opponents was shorter in post-conflict periods (PCs; ten minute focal samples on the initial recipient of aggression as soon as the conflict ceased) than during matched control periods (MCs; similar observations on the same individual at the same time the next day with no preceding aggression) (Kaplan–Meier survival analysis: χ^2^ = 11.299, df = 1, P = 0.001; Figure 1). The proportion of PC-MC pairs in which affiliation between former opponents occurred earlier in the PC than the MC or only the PC (‘attracted’ pairs; mean ±S.D.  =  0.195±0.16) was also significantly higher than the proportion of PC-MC pairs in which affiliation occurred earlier, or only in the MC (‘dispersed’ pairs; mean ±S.D. =  0.038±0.030; N = 6, t = 2.672, P = 0.044). For purposes of comparison with other populations and species, the mean (±S.D.) individual corrected conciliatory tendency (CCT; see Materials & Methods) was calculated as 0.16 (±0.14).

### When does reconciliation occur?

Reconciliation (defined as here as post-conflict affiliation between former opponents within ten minutes of the end of the preceding conflict) occurred after 37 of 197 conflicts. We investigated the effects of kinship, levels of contact sitting and preening, opponent sex-combination and conflict intensity on the occurrence of reconciliation using generalized linear mixed models (GLMMs; see Materials & Methods) in order to determine which characteristics influence the occurrence of reconciliation. Consistent with findings across the primate literature [18], we found support for the valuable relationship hypothesis as conflicts among kin were more likely to be reconciled than among non-kin (GLMM: β = 1.184; S.E. = 0.585; z = 2.024; P = 0.043). Moreover, we found that even when controlling for kinship, birds who were more likely to preen each other and sit in contact, characteristics previously shown to be related to high relationship value in ravens [17], were more likely to reconcile (β<0.001; S.E.<0.001; z = 3.254; P = 0.001). Neither the sex-combination of the opponents nor the intensity of the conflict affected the occurrence of reconciliation.

### The function of reconciliation

**Figure 2:**
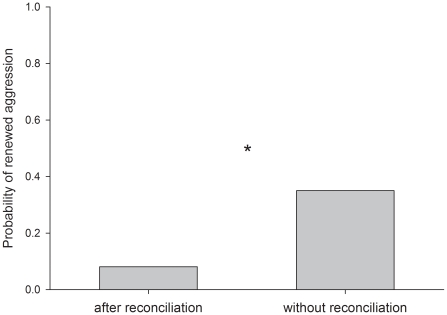
The probability of renewed aggression between former opponents during the post-conflict period after and without the occurrence of reconciliation. *P<0.05.

We found that renewed post-conflict aggression between former opponents was less likely to occur after than without reconciliation (χ^2^ = 10.359, df = 1, P = 0.001; Figure 2), but that reconciliation was not less likely to occur after than without renewed aggression (χ^2^ = 1.117, df = 1, P = 0.278), supporting the relationship repair hypothesis for the function of reconciliation in ravens. Moreover, we showed that when reconciliation does not occur, neither kinship (β<−0.229; S.E. =  −0.483; z = −0.475; P = 0.635) nor levels of contact sitting and preening (β<0.001; S.E.<0.001; z = −0.998; P = 0.318) influenced the occurrence of renewed aggression. This suggests that the interdependency between reconciliation and renewed aggression is not merely because valuable partners, who are more likely to reconcile, are less likely to engage in renewed aggression.

## Discussion

The apparent absence of reconciliation in birds has been suggested to result from a general difference in avian and mammalian behavior, possibly because the fluidity of avian social systems facilitates post-conflict dispersal [14], [15]. Aggressive conflict damages the opponents' relationship, leading to a loss of benefits afforded by the relationship, and results in post-conflict distress [19]. Reconciliation repairs the opponents' relationship and reduces post-conflict distress, but also entails risks of renewed aggression [3]. Post-conflict dispersal may thus offer a lower risk strategy. Moreover, while reconciliation may promote group cohesion vital for survival in the many primate species in which reconciliation occurs [3], such mechanisms may not be necessary in avian societies. Alternatively, the pair-bonded nature of most bird species may preclude the need for reconciliation as pair partners rarely engage in aggressive conflict [16] and other partners may not share a relationship of sufficient value to merit reconciliation. Our demonstration of reconciliation in this study, however, suggests that reconciliation can, and does, occur in (at least some) birds.

Ravens, although generally characterized by a pair-bonded society, may delay pair formation until at least their fourth year [20], and occasionally until as late as their tenth year [T. Bugnyar, unpublished data]. During the period between reaching independence and territorial pair formation, ravens may form large non-breeder flocks, enabling them to compete with territorial pairs for monopolisable food resources [21], [22]. Although the exact nature of such groups (e.g. consistency of group membership, relationships among group members) is unknown, it is likely that valuable relationships among non-kin and non-pair partners exist. Indeed, valuable relationships, characterized by a high frequency of allo-preening and agonistic support, have been shown to occur outside the pair bond among unrelated subadults in another captive raven colony (hereafter referred to as the ‘Austrian ravens’ as they were housed in the Austrian Alps) [17]. While reconciliation was not formally demonstrated in that population, in contrast to a study on rooks, where not a single case of post-conflict affiliation between former opponents was observed [14], the Austrian raven former opponents affiliated after 16 of 152 conflicts [15]. Thus, the difference between the Austrian ravens and the current study group may not be in whether they reconcile, but rather in the rate at which reconciliation occurs. This may reflect differences in observation effort and/or differences in the quality of their relationships (and thus in the costs and benefits of reconciliation). The latter would be consistent with findings in chimpanzees, where variation in conciliatory tendencies across populations is particularly evident and has been attributed to the plasticity of the nature of chimpanzee social relationships [3], [23].

Our findings indicate that the quality of the opponents' relationship is critical in determining whether reconciliation will occur in ravens. Furthermore, at least under certain conditions, ravens have relationships with partners of sufficient value to merit reconciliation and engage in aggressive conflict with such partners. The importance of relationship quality highlights the need for an accurate measurement of how the subjects assess their relationships with others, although exactly how to do this is a matter for debate [4], [24], [25]. In this study, we used kinship and levels of contact sitting and preening as indicators of relationship value, based on the assessment of relationship quality in another raven population [17]. However, as group members are likely to judge the quality of their relationships based on many different types of interactions, future studies should consider including a measure of relationship value that incorporates a wider range of ‘valuable’ behaviors, such as agonistic support and food sharing, data which were unavailable for this population, to improve the strength of the conclusions drawn.

It should be noted that the subjects of this study were captive ravens and, as very little is known about the composition of wild groups of subadult ravens, the likelihood of such relationships occurring in the wild and thus the likelihood of reconciliation in the wild is unclear. However, preliminary data on a non-breeder flock of wild ravens suggest that patterns of interactions indicative of valuable relationships, such as coalition formation, and patterns of aggression show a striking similarity to such patterns in aviary ravens with a similar group composition to the current study subjects (T. Bugnyar, unpublished data). Although reconciliation in primates has been suggested to be an artifact of captivity, a detailed analysis of rates of reconciliation across many primate species in the wild and in captivity has found no evidence to support this hypothesis [26]. Thus, while additional data on wild ravens is imperative, reconciliation does at least form part of their behavioral repertoire and may play an important role in the way in which they manage conflicts.

Reconciliation in primates has been shown to repair damage caused to the opponents' relationship by the preceding conflict and reduce post-conflict distress [3]. As no behavioral measures of distress have thus far been validated in birds, we were unable to test the distress-alleviation function during this study. However, although the limited sample size precluded us from conducting analyses at the individual level, we found that renewed aggression between former opponents was less likely to occur after reconciliation took place, suggesting that, as in primates, a primary function of reconciliation in ravens is to repair the relationship between valuable partners. Our findings could also be consistent with the hypothesis that reconciliation only occurs once hostility between former opponents has subsided, and thus partners with continued hostility do not reconcile, or with the hypothesis that reconciliation functions as a symbol of ‘benign intent’ [27]. However, as partners likely to share a more valuable relationship were more likely to reconcile conflicts, despite an equal chance of renewed aggression, and as subsidence of hostility (and thus subsequent reconciliation) is in itself suggestive of relationship repair, damage caused to the relationship by aggressive conflict appears to be mitigated following reconciliation, even if causality is not demonstrated.

Taken together, our results indicate that despite differences in social structure and evolutionary history, ravens exhibit similar conflict resolution strategies to primates, as former opponents engaged in post-conflict reconciliation to repair valuable relationships and reduce the likelihood of renewed aggression. Primate sociality has been suggested to differ from those of other mammals and birds because it is based on bonded relationships of a type that only exist in pair bonds in other taxa [16], [28]. Our findings suggest that such relationships may exist even *outside* the pair bond in ravens and that such partners may employ the same mechanisms in some birds as in primates to ensure that the benefits afforded by their relationships are maintained even when conflicts of interest escalate into aggression.

Recent research has unveiled that corvids may be capable of a whole host of cognitively demanding tasks that were previously considered to be the exclusive domain of apes and other primates, such as episodic-like memory [29], planning for the future [30], cooperative problem solving [31], creating novel tools to solve problems [32], and tactical deception [33]. The divergent evolutionary history and anatomical differences in brain structure between apes and corvids suggest that such similarities in cognition and behavior result from a convergent evolution of intelligence [34], [35], although little is yet known about the selection pressures driving the evolutionary processes in either group [36]. However, much of the focus of comparative social cognition has thus far been on experimental studies testing specific cognitive abilities rather than naturally occurring socially, and most likely cognitively, complex interactions, such as post-conflict behavior. This study, therefore, provides valuable further support for a convergent evolution of social strategies, in addition to mental processes, in avian and mammalian species in general, and in corvids and apes in particular.

## Materials and Methods

### Ethical Statement

All procedures were conducted in accordance with US law on animal research and treatment. Permits for ravens include US Federal Fish and Wildlife Permit Number MB689376-0, State of Maine Department of Inland Fisheries and Wildlife Permit 22077, and Vermont Fish and Wildlife Department Scientific Collecting Permit. Permission was received from the University of Vermont to observe the ravens for this study.

### Subjects and Housing

The study subjects were seven hand-raised ravens housed together in a 100 m^2^ outdoor aviary at the University of Vermont, USA. Six subjects from two nests (sibling group one: three males and a female; sibling group two: one male, one female) hatched in 2002, the seventh subject was an unrelated adult male hatched in 1999.

### Data collection

Data were collected from May 2002 to August 2003 by TB. All occurrences of aggressive conflict (defined as chase-flight, hitting or forced-retreat) were recorded along with the identities of the aggressor and victim (defined as initial recipient of aggression) and the intensity of the conflict (high = hit and/or ≥5 chase flights, low = forced retreat and/or <5 chase flights). The established post-conflict (PC)- matched control (MC) method [37] was used to collect post-conflict and baseline data. Each PC was a 10 minute focal sample on the conflict victim recording all affiliative (defined as contact sitting, preening or beak-to-beak or beak-to-body touching) and aggressive interactions, taken immediately after aggressive conflict ceased. MCs were similar observations on the same individual at the same time the next possible day. If the focal individual was involved in aggressive conflict in the ten minutes prior to the scheduled MC time, the MC was postponed for up to an hour after the time the PC was taken, or until the following day. PCs were abandoned if no MC was recorded within a week of the initial conflict. Data on the quality of social relationships were obtained through 57 30-min all occurrences group samples spread across the data collection period, which enabled us to calculate the proportion of time each possible dyad combination spent sitting in contact or preening each other. In a previous study of relationship quality in ravens, these two behaviors were found to be indicative of high relationship value as they associated strongly with agonistic support (a clearly beneficial behavior), but not with variables thought to represent simply compatibility or tolerance (level of aggression, response to approaches, counter-intervention) [17]. Thus, we used the total proportion of time spent contact sitting or preening per dyad as a measure of the value of their relationship.

### Data analysis

Data analysis was based on 197 PC-MC pairs on six subjects (range 17–46 pairs per individual). The only adult subject was never recorded as a conflict victim.

In order to determine whether reconciliation occurred, the mean proportion of PC-MC pairs per individual in which affiliation between former opponents occurred only in the PC, or earlier in the PC than the MC (‘attracted’ pairs) was compared those in which such affiliation occurred earlier, or only, in the MC (‘dispersed’ pairs) using a paired t-test. The latency to first affiliative contact between former opponents in PCs and MCs was also compared using a Kaplan–Meier survival analysis with a Mantel–Cox test. We did not look for selective attraction between former opponents as ravens engage in frequent post-conflict affiliation with bystanders uninvolved in the preceding conflict [15]. We used the Corrected Conciliatory Tendency (CCT) to calculate a measure of mean rate of reconciliation controlling for baseline levels of affiliation using the following formula: (number of attracted pairs – number of dispersed pairs)/total number of PC-MC pairs [38].

We used generalized linear mixed models (GLMMs) to investigate the effects of conflict intensity (high or low), opponent sex combination (male-male, male-female, female-female), kinship (sibling or non-sibling) and proportion of time spent contact sitting or preening on the occurrence of reconciliation (yes/no). An additional GLMM was run investigating the effects of kinship and proportion of time spent contact sitting or preening on the occurrence of renewed aggression (when reconciliation did not occur). For all GLMMs, the identities of both conflict opponents were entered as random factors. We used GLMMs with binomial error structures and a logit-link function. Akaike's information criteria (AIC) values were used to select the best (most parsimonious) model [39]. Only the effects of variables remaining in the best model are presented, except where none of the independent variables was found to significantly influence the dependent variable, in which case the effects of all independent variables are presented.

To examine the interdependency reconciliation and renewed aggression between former opponents, we compared the probability of renewed aggression occurring after and without reconciliation using a Chi^2^ test.

GLMM analyses were conducted using the lme4 package [40] in R (www.r-project.org). All other analyses were conducted in SPSS v.17. Data conformed to normality whenever parametric tests were used. All tests were two-tailed and the alpha level was set at 0.05.
